# BAMQL: a query language for extracting reads from BAM files

**DOI:** 10.1186/s12859-016-1162-y

**Published:** 2016-08-11

**Authors:** Andre P. Masella, Christopher M. Lalansingh, Pragash Sivasundaram, Michael Fraser, Robert G. Bristow, Paul C. Boutros

**Affiliations:** 1 Ontario Institute for Cancer Research, Suite 510, 661 University Avenue, M5G 0A3, Toronto, Canada; 2 Ontario Cancer Institute, Princess Margaret Cancer Centre/University Health Network, Toronto, Canada; 3 Department of Pharmacology & Toxicology, University of Toronto, Toronto, Canada; 4 Department of Medical Biophysics, University of Toronto, Toronto, Canada; 5 Department of Radiation Oncology, University of Toronto, Toronto, Canada

**Keywords:** BAMQL, Query language, BAM-format

## Abstract

**Background:**

It is extremely common to need to select a subset of reads from a BAM file based on their specific properties. Typically, a user unpacks the BAM file to a text stream using SAMtools, parses and filters the lines using AWK, then repacks them using SAMtools. This process is tedious and error-prone. In particular, when working with many columns of data, mix-ups are common and the bit field containing the flags is unintuitive. There are several libraries for reading BAM files, such as Bio-SamTools for Perl and pysam for Python. Both allow access to the BAM’s read information and can filter reads, but require substantial boilerplate code; this is high overhead for mostly ad hoc filtering.

**Results:**

We have created a query language that gathers reads using a collection of predicates and common logical connectives. Queries run faster than equivalents and can be compiled to native code for embedding in larger programs.

**Conclusions:**

BAMQL provides a user-friendly, powerful and performant way to extract subsets of BAM files for ad hoc analyses or integration into applications. The query language provides a collection of predicates beyond those in SAMtools, and more flexible connectives.

**Electronic supplementary material:**

The online version of this article (doi:10.1186/s12859-016-1162-y) contains supplementary material, which is available to authorized users.

## Background

Binary Alignment/Map (BAM) provides a common format to hold large quantities of genomic read data after alignment to a reference genome. Reads are annotated with supplementary information not present in FASTA or FASTQ files, such as target position and chromosome, and information about the mate pair.

Often, only a subset of reads are needed for further analysis. SAMtools, the standard software for manipulation of BAM files, provides options to perform limited filtering of BAM files and collect matching reads [1]. The interface is neither expressive nor user-friendly: the user must indicate which bit flags they require not using names, but the numeric values of those flags. Further, the selection condition is restricted to a filter that describes which bit flags must match, which must not, a minimum quality score and a list of matching read groups. This limits query expressiveness; for example, selecting reads with poor quality is impossible.

General purpose programming languages can be used with BAM file manipulation libraries to filter a file. Since most subsetting operations are ad hoc, this is cumbersome and requires substantial boilerplate code. More concerning, there is a disparity between the checking done by the language and what is needed by the BAM APIs. For instance, it is possible to read only a subset of a file using an index, but this requires correctly matching chromosome names between the index request and the filtering expression. A mismatch may result in a silent omission of data that is difficult to detect due to the complex, mixed-language query.

To simplify the subsetting process, but retain the ability to have powerful queries, we developed BAM Query Language (BAMQL), a domain-specific language for matching BAM reads.

## Implementation

BAMQL queries are specified by predicates and logical connectives. The predicates include ones which match BAM flags, mapping information, auxiliary read information, position and the sequence. For example, to output a BAM file with all reads which are on chromosome 1 and have a mate read, one could do the following: bamql -f input.bam -o output.bam ’chr(1) & paired?’. An outline of all available connectives and predicates can be found in Table 1.

**Table 1:** Descriptions of BAMQL connectives and predicates

Category	Syntax	Description
Logical Connectives	! expr	Is satisfied if expr is not satisfied.
	expr | expr	Is satisfied if at least one operand is satisfied.
	expr ^ expr	Is satisfied if at exactly one operand is satisfied.
	expr & expr	Is satisfied only if both operands are satisfied.
	cond then then_expr else else_expr	If cond is satisfied, this expression will only be satisfied if then_expr is satisfied. If cond is not satisfied, then this expression will only be satisfied if else_expression is satisfied.
BAM Flags	paired?	The read is paired in sequencing.
	proper_pair?	The read is mapped in a proper pair
	unmapped?	The read is unmapped.
	mate_unmapped?	The mate is unmapped.
	mapped_to_reverse?	The read is mapped to the reverse strand.
	mate_mapped_to_reverse?	The mate is mapped to the reverse strand.
	raw_flag(int)	The read matches the specified SAM flag.
	read1?	The read is the first read in a pair.
	read2?	The read is the second read in a pair.
	secondary?	The alignment is not primary.
	failed_qc?	The read failed platform/vendor quality control checks.
	duplicate?	The read is either a PCR or optical duplicate
	supplementary?	The alignment is supplementary.
Mapping Information	chr(glob)	The read is mapped to the specified chromosome.
	mapping_quality(probability)	Matches the read if the proability of error is less than the specified probability.
	mate_chr(glob)	The mate is mapped to the specified chromosome.
	split_pair?	Matches if both the read and it’s mate pair are mapped, but only if to different chromosomes.
Position	after(position)	Matches all reads that cover the specified position or any higher position.
	before(position)	Matches all reads that cover the specified position or any lower position.
	position(start,end)	Matches all sequences that cover the range of position from specified start to end, i.e. N matches any base.
Sequence	nt(position,n)	Matches all reads that are classified as the IUPAC-style nucleotide n at the specified position, i.e. N matches N.
	nt_exact(position,n)	Matches all reads that exactly match the IUPAC-style nucleotide n at the specified position.
Miscellaneous	read_group(glob)	Matches the read group.
	header ~ /regex/	Matches a Perl-compatible regular expression against the read’s header.
	random(probability)	The read is chosen based on a uniform pseudo-random variable.
	true	Always satisfied.
	false	Never satisfied.

BAMQL has a library which compiles a query into native code, then checks the reads in the input BAM file, saving the matched reads to a user-specified output BAM file. A recursive descent parser reads the query and the resulting structure is converted into native code using LLVM 3.4.0 or later [2]. A driver program reads the input BAM file, checking each read against the query and tracking the number of accepted and rejected reads, which it then writes to BAM files. All BAM file operations are done using HTSlib 1.3 [1]. The command line interface uses filename specifiers for input and output so, when utilizing pipes in the bash environment, /dev/stdin and /dev/stdout will allow for writing across pipes.

If an indexed BAM is provided, the query automatically determines if only a subset of the file needs to be analysed to improve performance. SAMtools has the same performance gain by specifying regions, but, there is the possibility of mismatching the regions specified and those in filter.

## Results

To test the efficacy, we compared several equivalent queries written in BAMQL, SAMtools (v1.3) + GNU AWK (v4.0.1), Sambamba (v0.5.9), Python using pysam (v0.8.2) [3], Perl using Bio-SamTools (v1.41) [4], and C using HTSlib (v1.1). We compared execution times and the quantity of code written. Execution time is an imperfect metric, as it is dependent on various properties of the system at runtime, such as CPU load, disk operations, and the hardware specifications of the system. By keeping these factors equivalent when performing benchmarks, we can see relative differences between the tools. All tests are provided in the additional files [see Additional file 1] and examples of queries and a list of predicates can be found on the github wiki page [see Availability of data and materials]. Six tests were defined:

- **all** – copies the input to establish a baseline I/O cost.
- **paired** – keeps only reads which are paired.
- **chrY** – keeps reads on the Y chromosome, whether it is named “Y” or “24”, optionally prefixed with “chr”, irrespective of case.
- **mt** – keeps all reads which they or their mate pair are unmapped or mapped to the mitochondrial genome (named “M”, “MT” or “25”, optionally prefixed with “chr”, irrespective of case).
- **nt** – keeps all reads which have base C at position 13353 of the reference genome after alignment.
- **chain** – filters chromosomes 1, 2 and 3 into separate BAM files.

The tests were written as simply as possible. No profiling or intentional optimisations were done as this test code is meant to be a single-use ad hoc query.

### Method-comparison

It must be noted that *nt*, *mt* and *chrY* tests could not be written with SAMtools alone and required output to be piped through AWK scripts for the actual filtering process. Similarly, Sambamba required an AWK script to implement filtering for the *nt* test.

The difference in user-friendliness between BAMQL and SAMtools is evident in the relatively simple *chrY* test. The BAMQL query for a *chrY* test (listing ??) is a one-line query since BAMQL recognizes the differing names for the human Y chromosome and checks them automatically.



The same query implemented using SAMtools (listing ??) however requires the query to be run using an AWK filter in order to manually check for the multiple ways Y chromosome could be labelled in the BAM file.



Sambamba was more expressive than SAMtools in this case. However, Sambamba requires chromosomes to be specified using reference ID rather than chromosome name [5]. Sambamba’s expressiveness also do not extend to complex queries such as the *chain* test that requires chaining filters. While BAMQL had a dedicated function for handling a chain of filters, each with an optional output file, Sambamba and SAMtools both required separate queries for each of the filters.

A summary of the differences in capabilities of the tools used for testing is shown in Table 2.

**Table 2:** Comparison of the filtering capabilities of the various tools

Tool	Dedicated filtering	Supports multiple filters	Supports logical connectives	Supports chaining filters	Filters reads by mapping	Filters reads by flags	Filters reads by sequence
BAMQL	✓	✓	✓	✓	✓	✓	✓
C	✗	✓	✓	✓	lim	✓	✓
Perl	✗	✓	✓	✓	lim	✓	✗
Python	✗	✓	✓	✓	lim	✓	✗
SAMtools	✓	✗	✗	✗	lim	lim	✗
Sambamba	✓	✓	lim	✗	lim	✓	✗

### Performance

All tests were run three times on an Intel Core i5 3.4 GHz Ubuntu 14.04 workstation with a 10 GB indexed, sorted BAM file containing male human whole exome sequence as input. The reads from each implementation were compared, and were identical in all cases. The coefficient of variation of user time was < 4 % while that of system and wall times were noisier, up to 15 %.

The *mt* and *nt* tests, shown in Tables 3 and 4 respectively, are more processing-intensive while the *paired* and *chain* tests, shown in Tables 5 and 6 respectively, are less processing-intensive and keeps more sequences, causing it to be more output-intensive. Two notable outliers are Perl and Sambamba. Perl consumes much more CPU time than the other tools; this is evidenced by high user times in the *mt* test. Tests implemented with Sambamba alone, *paired* and *mt*, have a low wall time due to parallel processing capabilities of Sambamba. However, Sambamba+AWK and SAMtools+AWK have high system times, due to the overhead to cope with pipe operations. C, Python and Perl seem to incur a high overhead in the *chain* test due to multiple output files opened for depositing data. Overall, BAMQL, C and Python performed well. In the output-dominated tests, *paired* and *chain*, the differences in real processing become clouded by the cost of writing the output. Notice that the user and wall times show a smaller percentage difference in *paired* compared to *mt*. In the more logic-intensive test, *mt*, the languages separate out more.

**Table 3:** Comparison of performance for selecting mitochondrial sequences (*mt*)

	System	User	Wall	Code
Tool	time (s)	time (s)	time (s)	(bytes)
BAMQL	**3.65**	133.86	150.28	**86**
C	3.80	**126.28**	138.16	1315
Perl	4.57	1704.05	1716.72	498
Python	4.06	299.33	313.68	507
SAMtools+AWK	14.56	455.12	240.96	170
Sambamba +AWK	6.53	185.64	**99.56**	112

Best performer is highlighted in bold

**Table 4:** Comparison of performance for selecting reads with base at a sequence position (*nt*)

	System	User	Wall	Code
Tool	time (s)	time (s)	time (s)	(bytes)
BAMQL	**3.55**	109.16	115.69	**54**
C	2.89	**107.49**	**114.17**	2590
Perl	3.73	264.84	356.29	1667
Python	4.24	137.06	171.77	1225
SAMtools+AWK	41.42	2591.00	2322.19	831
Sambamba+AWK	56.12	3446.93	3100.96	874

Best performer is highlighted in bold

**Table 5:** Comparison of performance for selecting paired-end sequences (*paired*)

	System	User	Wall	Code
Tool	time (s)	time (s)	time (s)	(bytes)
BAMQL	**22.54**	**1271.20**	1364.64	**45**
C	29.31	1277.55	1377.29	706
Perl	28.54	1383.65	1474.48	319
Python	29.48	1314.25	1413.04	262
SAMtools	28.26	1271.97	1382.81	46
Sambamba	35.11	1855.81	**583.02**	53

Best performer is highlighted in bold

**Table 6:** Comparison of performance for applying chain of filters to separate by chromosomes (*chain*)

	System	User	Wall	Code
Tool	time (s)	time (s)	time (s)	(bytes)
BAMQL	**4.83**	305.66	345.19	**123**
C	9.31	385.05	422.02	1027
Perl	11.18	2552.69	2585.88	800
Python	11.18	576.44	643.36	907
SAMtools	6.71	**302.15**	329.63	156
Sambamba	8.45	441.58	**145.49**	174

Best performer is highlighted in bold

With the exception of the *nt* test, the BAMQL implementation is the most performant with 24 % to 90 % faster system time than the C implementation. In terms of code volume, BAMQL required much less code; typically half of the SAMtools+AWK and a tenth of the C implementation. An additional csv file shows the compete results for all tests [see Additional file 2].

## Discussion

Since most BAM filtering operations are ad hoc, the runtime performance of BAMQL is not especially important, although it outperforms most standard approaches. We believe that BAMQL’s major impact is the simplicity of the queries and the automatic guards against common mistakes. In particular, BAMQL generates index requests from the BAM and does the filtering using the same query. This guards against accidental mismatches in chromosome names caused by independent index requests and filtering expressions (*e.g.* SAMtools with AWK filter). It also automatically corrects for common chromosomal name irregularities. These features make it easy to write correct queries and, by reducing boilerplate, makes them simple to read and understand.

Since the performance of BAMQL queries is good, it is also possible to use them in other contexts. Queries can be compiled to native machine code, making it trivial to integrate into a larger C or C++ program. There are queries that are outside the domain of BAMQL: BAMQL queries are stateless, while general purpose languages are not. Given the low overhead of integration, it is reasonable to split a stateful query between C or C++ and BAMQL.

## Conclusions

BAMQL is designed to improve the efficacy and user-friendliness of the current tools like SAMtools and Sambamba. It provides a larger collection of predicates than SAMtools or Sambamba. These predicates can be joined together with a wider set of logical connectives, allowing for more expressive queries. By compiling queries into native code using LLVM, BAMQL is also able to perform as well as C but with significantly simpler code. BAMQL provides a powerful yet intuitive approach to ad hoc querying BAM formatted data.

## Availability of data and materials

The complete test-suite as well as results supporting the conclusions of this article are included as additional files. The test-suite is also available on the homepage. Ubuntu packages for the latest version of BAMQL are available.**Project Name:** BAMQL **Project home page:**https://labs.oicr.on.ca/boutros-lab/software/BAMQL**Version:** 1.1 **Operating System:** Platform independent **Programming language:** C++, C **Other requirements:** LLVM 3.4.0 or higher, CLANG, HTSlib and libuuid are required to compile**License:** MIT **Any restrictions to use by non-academics:** None

## Abbreviations

BAM, binary alignment/map; BAMQL, BAM query language

## Additional files

Additional file 1 This directory contains tests written in BAMQL, C, Perl, Python, SAMtools and Sambamba languages. A binary file with the appropriate version is provided for Sambamba. (ZIP 1495 kb)

Additional file 2 This file contains the complete results of the test-suite in a csv format. (XLS 32 kb)
